# Pilot Study on Gaze Characteristics of Older Drivers While Watching Driving Movies

**DOI:** 10.3390/geriatrics9050132

**Published:** 2024-10-10

**Authors:** Kaori Kawabata, Yuya Nakajima, Kazuki Fujita, Mamiko Sato, Koji Hayashi, Yasutaka Kobayashi

**Affiliations:** 1 Graduate School of Health Science, Fukui Health Science University, Fukui 910-3190, Japan; k.fujita@fukui-hsu.ac.jp (K.F.); satomoko@fukui-hsu.ac.jp (M.S.); yasutaka_k@fukui-hsu.ac.jp (Y.K.); 2 Fukui Higher Brain Dysfunction Support Center, Fukui 910-0067, Japan; y.nakajima@fukui-hsu.ac.jp; 3 Department of Rehabilitation, Faculty of Health Science, Fukui Health Science University, Fukui 910-3190, Japan; 4 Department of Rehabilitation Medicine, Fukui General Hospital, Fukui 910-8561, Japan; kjhayashi@f-gh.jp

**Keywords:** older driver, hazard prediction, gaze analysis

## Abstract

**Objective**: This study aims to clarify the gazing characteristics of older drivers while driving cars using a gaze analysis device. **Methods:** The participants included 16 older and 12 middle-aged drivers who drove cars daily. After conducting cognitive and attentional function tests, eye gaze while watching driving videos was measured using an eye tracker. Ten driving videos were prepared. In addition, a total of 34 hazard areas were analyzed. **Results:** The results of the gaze measurement parameters were statistically compared between the two groups. In the older group, the gaze analysis results indicated that while viewing driving videos, the search for areas close to the car was expanded. In addition, in several hazard areas, we observed a decrease in the number of drivers gazing at the driver, shortened total gazing time, delay in the timing of gazing, and decrease in the number of visits. **Conclusions**: Older drivers’ eye movement is increased; however, it is characterized by gazing at unimportant areas, indicating an inefficient scanning pattern. Although these results do not indicate an obvious decline in driving ability among older drivers, the decline in hazard perception may become apparent in some situations. The data contain underpowered results and require revalidation in larger studies.

## 1. Introduction

According to the Traffic Accident Situation in Japan [1], the number of traffic crashes per 100,000 license holders by age group of drivers of mopeds and larger vehicles is highest among those aged 16–19 years, followed by those aged 20–24 years, and those aged 85 years and older. The crash rate by age group shows a decrease after the age of 30 and an increase after the age of 70. In addition, the number of fatal crashes per 100,000 license holders by age group for drivers of mopeds and larger vehicles is higher for those aged 70 years and older than for those aged 16–24 years [1]. In other words, although traffic crashes are more frequent in Japan among young drivers aged 16–24 years and older drivers aged 70 years and older, the crash rate for young drivers declines with age and increased driving experience. In comparison, the crash rate increases for older drivers who are also more likely to cause serious traffic crashes. Thus, traffic crashes by older drivers have become a serious social problem, leading to stricter licensing policies for older drivers in Japan, encouraging them to voluntarily return their driver’s licenses. However, automobiles are essential in daily life, especially in automobile-dependent areas. Moreover, owing to the aging population as well as depopulation, driving is vital to maintaining the lifestyle of older adults. When older adults stop driving, their health is affected, which includes decreased social participation, depression, cognitive decline, and an increased risk of nursing home placement and death [2,3,4,5,6,7]. Furthermore, being forced to stop driving may lead to the use of higher-risk modes of transportation (e.g., walking or cycling), which may result in traffic accidents [8]. Given the health and social problems that can be prevented by continuing to drive, efforts should be made to help older drivers continue to drive safely.

It has been estimated that 95% of traffic crashes are caused by human error [9,10], and one of the most basic and important skills a driver should possess is the ability to anticipate danger [11,12,13]. Horswill and McKenna [14] describe hazard prediction as an individual’s ability to anticipate potentially dangerous situations on the roads. Hazard prediction is an important factor in determining the crash risk for older drivers [15]. Quimby and Watts [16] have indicated that hazard prediction is fastest among skilled middle-aged drivers (35–54 years old), reaches its peak at age 55, and declines thereafter. As hazard prediction is an ability that improves with driving experience, and many older drivers have sufficient driving history, lack of experience is rarely a barrier [17]. As cognitive functions including processing speed and situational awareness decline for those over 65 years of age, they experience difficulty compensating for neurodegeneration, and their danger prediction during driving may be reduced [18,19].

According to a systematic review of driving hazard perception tests [20], hazard perception tests come in a variety of forms, including questionnaires, fixed images, film or footage, simulators, and driving tests. Although there is positive evidence regarding the effectiveness of driving simulators in predicting on-road driving performance [21,22], the use of simulators has been shown to cause problems with simulator sickness, especially in older adults [23,24]. Omran et al. [20] have reported that static tests using questionnaires or fixed images fail to replicate the dynamic nature of real-world driving and may lack ecological validity when measuring hazard perception; therefore, film or video may be more appropriate for testing driver hazard perception. Hazard prediction ability has been assessed using reaction time measurements [17,25,26,27], eye gaze [28,29,30], and verbal commentary [18,19]. A study on hazard prediction ability in drivers aged 65 years and older found a significant relationship with crash history [17], delayed reaction time to hazards [25,28], decreased hazard discrimination [30], and decreased situational awareness [19]. This finding is inconsistent, however, and older drivers are reported to be as safe as younger drivers. Therefore, Hakamies-Blomqvist et al. [31] have hypothesized that there is no difference in scanning behavior when viewing driving videos, and Underwood et al. [27] and Borowsky et al. [25] have found no evidence of age-related decline in the search for scenes of danger detection. Many studies on hazard prediction and eye tracking have focused on young drivers with limited driving experience. Inexperienced drivers with poor hazard prediction have been reported to detect fewer hazards [32,33] and fixate fewer times [34]; however, inexperienced drivers’ fixation times are longer and their cognitive processing speed is slower [35]. Contrastingly, experienced drivers with excellent hazard prediction show efficient scanning patterns, shorter cumulative gazing times [36,37,38], and frequent gazing at hazard lurking points [39]. 

Research specific to older adults’ driving is still limited [40,41]. Depestele et al. (2020) [42] reported a literature review of 22 studies on older adults’ driving performance using driving simulators. The review found that older drivers exhibit greater variability in speed, distance between vehicles, and lane-keeping behavior, as well as inconsistent driving performance. Declines in executive function, attention, dual task performance, visuospatial ability, processing speed, and memory—all indicators of age-related cognitive decline—can negatively impact daily activities such as driving [43,44,45,46,47]. Particularly, executive function and attention are considered essential for driving, and these cognitive functions have been shown to decline with age [48,49,50]. Moreover, with age, there are changes in brain structure and connectivity, namely reduced gray matter volume and white matter tracts, possibly due to disruption of integrity [51,52,53]. These cognitive impairments are likely to result in diminished driving skills; however, Depestele et al. (2020) [42] noted the paucity of research on vision, including visuospatial cognition. Research on vision in older adults suggests that they are often slower and less accurate in their visual search, indicating age-related declines in visual attention [54], efficiency of the search process [55], and visual cognition [56]. The decline in older adults’ performance in visual search is not solely attributable to bottom-up processing; it is also partly due to reduced top-down attentional control [54]. Furthermore, with respect to eye movements in older adults, performance of most eye movements, including fixation and tracking, is thought to be reduced [57,58]. The horizontal and upward range of eye movements is more likely to decrease, while the downward range is more likely to be maintained [59,60].

A literature review of eye tracking in older drivers [40] has found that they have reduced hazard detection [25], reduced scanning behavior [28], perform fewer checks of traffic conditions when turning right or left [29], and experience difficulty identifying the view at night [61]. This review [40] suggests that inconspicuous vehicles (e.g., motorcycles) [62], pedestrians in more complex urban environments [37], auditory stimuli [63], and even electronic billboards [64] can result in late fixation during eye tracking driving tasks, indicating a possible increase in crash risk. It has been suggested that gaze may be delayed during the task, potentially increasing the risk of collision. Several studies have reported that older drivers do not pay attention to hazards present in the driving environment or the actions of others, focus on less dangerous stimuli [18], and have a narrow scanning range at intersections [23]; however, few studies focus on the hazard prediction and gazing characteristics of older drivers. Older adults have been shown to drive at slower speeds in more complex driving situations [45,65,66,67]. It is speculated that these behaviors are intended to compensate for cognitive decline, reduced visual attention, and reduced efficiency of visual search. Driving is a complex skill that requires multiple abilities, including adequate visual, motor, and cognitive skills [68,69], and these aspects should be analyzed from multiple angles. No studies have focused on the aspects of hazard prediction and gazing characteristics while driving, and further research is needed to delve into the characteristics of older drivers.

The research question for this study was set as follows. “Do older drivers have different gazing characteristics when driving compared to middle-aged drivers?” Accordingly, this study aims to clarify the gaze characteristics of older drivers while driving a car using a gaze analysis device. Eye tracking technology has the potential to improve our understanding of driver scanning patterns and visual attention [70] and may help explain why older drivers have high crash rates. The main hypothesis of this study is that older drivers will be slower to detect hazards and more likely to miss potential hazards than middle-aged drivers. Furthermore, older drivers are expected to be less efficient in their visual search patterns, have a narrower gazing range, and take longer to scan the driving environment. We hypothesized that these factors would reduce their ability to anticipate and respond to hazards, ultimately reducing driving safety. While previous studies have mostly evaluated behavioral data such as reaction time and error rate, or task performance using a driving simulator, the novelty of this study is that it utilized eye tracking technology to conduct a detailed analysis of the gazing characteristics of older drivers to predict hazards. By conducting eye movement analysis during the viewing of driving videos, it was possible to assess natural visual attention while reproducing the driving scenario. Analysis of eye movements in free viewing tasks includes visualization of gaze patterns to elucidate top-down and bottom-up behaviors [41]. This approach provides a more direct understanding of the visual cognition characteristics in older adults, compared to traditional evaluations, which were based solely on reaction times and error rates. It provides a new methodology and is expected to contribute to the development of safe driver assistance systems and driver education programs. In an aging society, supporting older adults’ driving ability will help them maintain their health and improve their quality of life. Therefore, this study, which focused on hazard prediction and gazing characteristics, is significant in that it provides new knowledge that will be useful in developing strategies to maintain and improve the driving ability of older adults.

## 2. Materials and Methods

### 2.1. Recruitment of Participants

Participants were recruited in writing between March 2023 and February 2024 from areas in Japan with high automobile dependence. Inclusion criteria were older adults (70s) and middle-aged adults (30–40s) who drove daily and agreed to participate in the study after an oral and written explanation of the purpose and methods of the study was provided. Exclusion criteria were a driving history of less than one year, cognitive impairment (Mini-Mental State Examination [MMSE] score of 23 or less), and a history of neurological disease.

### 2.2. Creation of Driving Videos

The author drove within urban and residential areas and recorded driving videos using a drive recorder. From the recorded video, the author extracted and edited parts of the video that required attention and danger prediction and created a driving video. Microsoft Clip Champ was used to edit the videos. Each 40 s scene consisted of a 10 s video showing the driving directions, a 25 s driving video, and a 5 s plain video for breaks. The driving video included scenes such as entering an intersection, turning right or left at an intersection, and passing through a railroad crossing; scenes requiring consideration of approaching cars and oncoming traffic; and scenes involving pedestrians, bicyclists, and crossers.

### 2.3. Selection of Hazard Areas

A hazard is “any object, situation, occurrence, or combination of these that introduce the possibility of the individual road user experiencing harm” [71]. Hazard areas in the driving videos were selected as areas of interest (AOIs).

Ten middle-aged drivers with excellent hazard perception [16] and no crashes or violations in the past three years were asked to watch the driving video and point out situations to which they paid attention when driving. Collaborators’ age and driving experience were 41.6 years (SD = 4.6) and 22.6 years (SD = 4.7), respectively. The collaborators were instructed to watch the video as if they were driving themselves, stopping the video at any time to verbally describe any areas that may be potentially hazardous and require close attention while driving. A laptop computer (16-inch, Dell) was used to view the driving scenario. The collaborators’ remarks were recorded and extracted. Based on the recorded data, the AOIs were set at points identified by seven or more of the ten participants. Two to six AOIs were set for each scene, with a total of thirty-nine AOIs (Scene 1: AOI 1–5, Scene 2: AOI 6–8, Scene 3: AOI 9–12, Scene 4: AOI 13–15, Scene 5: AOI 16–18, Scene 6: AOI 19–20, Scene 7: AOI 21–23, Scene 8: AOI 24–28, Scene 9: AOI 29–33, and Scene 10: AOI 34–39). The AOI interval was defined as the time interval from the moment the hazard was first observed on the video until the car passed the hazard. The average time was 7.9 s (SD = 3.5).

### 2.4. Assessment Items

#### 2.4.1. Basic Attribute Questionnaire

Sex, driving frequency, driving time, and experience of traffic violations and crashes were assessed using questionnaires.

#### 2.4.2. Cognitive and Attentional Function Tests

The MMSE, Trail Making Test Part A (TMT-A), Trail Making Test Part B (TMT-B), and Symbol Digit Modality Test (SDMT) were performed.

#### 2.4.3. Eye Gaze Measurement during Driving Video Viewing

**Figure 1:**
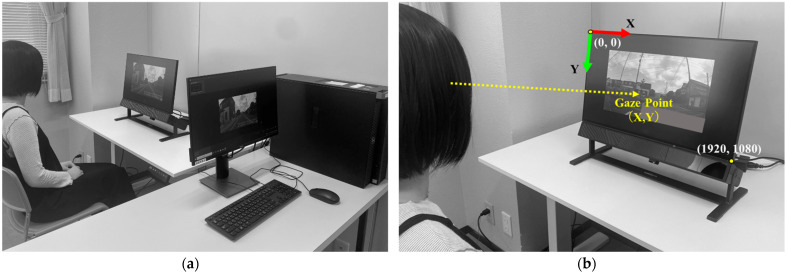
(**a**) Equipment and experimental environment; (**b**) coordinates of the gaze point.

Equipment and Experimental Environment (Figure 1a); Driving videos were presented on a 23.8-inch monitor (1920 × 1080 pixels) synchronized with an eye tracking device. The eye tracking device used in this study was a screen-based eye tracker (Tobii Pro Spectrum 60 Hz ver. 2.2.3, Tobii Technology, Inc., Shinagawa-ku, Tokyo, Japan). The biometric measurements and analysis software (Tobii Pro Lab Ver. 1.171; Tobii Technology, Inc.) was used to analyze gaze measurement data. The distance between the screen and the eyeballs was approximately 60 cm. Nine calibration points were used. An angular velocity of 30°/s or less was defined as fixation and an angular velocity of 30°/s or more was defined as saccade.

The participants were connected to an eye tracker and told that their eye movements would be recorded. They sat in front of the display, and the height of the monitor was adjusted so that the participants’ eyeballs were positioned at the center of the monitor in the chair seating position. The chair seat height was standardized to 43 cm. After a short calibration period, the participants watched a practice video to familiarize themselves with the experimental setup. The participants were asked to watch the driving video as if they were driving themselves. After viewing the driving video, participants were asked whether they could imagine driving using a Visual Analog Scale (VAS) to assess their subjective driving image level.

Eye Movement Data: The following parameters were obtained.

(a) Coordinates of the Gaze Point (X, Y): The upper left corner of the monitor is the origin (0,0), and the lower right corner is (1920,1080). In Figure 1b, the horizontal direction is indicated by the X-axis, and the vertical direction by the Y-axis. The average, maximum, and minimum values for each scene of the driving video were calculated. After each value for a total of 10 scenes was added and averaged, the values were converted from values representing coordinates (pixel) to percentages (%). Normally, hazards appear at various locations and times, and drivers must pay attention to road conditions comprehensively. By performing an additive average, we obtained general gazing coordinates that are not limited to specific road situations. The maximum value of the X coordinate indicates the rightmost, and the minimum value indicates the leftmost, representing the horizontal search range; the maximum value of the Y-coordinate indicates the bottommost and the minimum value indicates the topmost, representing the vertical search range. All coordinates for cases in which the subject was gazing at something other than the driving video were excluded.
(b) Total Number of Fixations: This is the number of gazing counts in the driving video (25 s) for each scene. A higher number of gazes indicates more eye movement.
(c) Percentage of Participants who Gazed at AOI: The percentage of drivers who gazed at the AOI was calculated. The value is involved in the hazard detection rate.
(d) Total Duration of Fixation in AOI: The total time spent gazing at the AOI was calculated. The duration of fixation was normalized at each AOI interval time because it depends on the time the hazard is represented [37]. For example, if the cumulative gazing time was 1.5 s in a 5.0 s AOI interval time, the normalized cumulative gazing time would be 30.0%.
(e) Time to First Fixation in AOI: This is the time it took to gaze at the AOI for the first time and it involves the speed of reaction to a hazard.
(f) Number of Visits in AOI: It is the number of revisits after leaving the AOI. The number of revisits was normalized by dividing by the number of gazing times calculated for “(b) Total Number of Fixations” [70]. For example, if the number of gazing times in “(b) Total Number of Fixations” is 50 and the number of visits to the AOI is 5, the normalized number of visits is 10.0%.

### 2.5. Analysis

Basic attributes, cognitive and attentional function test results, and eye movement data of the older and younger groups were compared statistically. Since the data included small expected values and data that did not follow a normal distribution, Fisher’s exact test and Mann–Whitney U test were used for comparisons. IBM SPSS Statistics 25 was used, and a 5% significance level was set. The measures of effect size (ES) were Cohen’s h for Fisher’s exact test and r for Mann–Whitney U test [72,73]. A posteriori power analysis was conducted using G*Power 3.1.9.7 to calculate power value [72].

## 3. Results

### 3.1. Participants

This study’s participants comprised 16 older and 11 middle-aged drivers (Table 1). All the participants lived in Japan in automobile-dependent areas. Age and driving history averaged 73.9 (standard deviation; SD 3.2) years and 49.1 (SD 9.7) years for the older group, and 37.3 (SD 2.9) years and 18.0 (SD 2.4) years for the middle-aged group, respectively. All the participants had normal or corrected vision. The number of participants who wore nude/contact lenses/glasses was 12/1/3 in the older group and 4/5/3 in the middle-aged group. Only two participants in each group had a history of eye surgery.

### 3.2. Basic Attributes

Table 2 presents the comparison results of the basic attributes. No significant differences were observed between the two groups regarding sex, driving frequency, driving time, experience of traffic violations, and crashes.

### 3.3. Cognitive and Attentional Functions

Table 3 shows the comparison results of the MMSE scores, TMT-A time required, TMT-B time required, and SDMT achievement rate. Significant differences were found in all the results, with the older group showing lower cognitive function, attention, and processing speed than the middle-aged group (MMSE: *p* = 0.002 ES(r) = 0.58 power = 0.93, TMT-A time required: *p* < 0.001 ES(r) = 0.72 power = 0.99, TMT-B time required: *p* < 0.001 ES(r) = 0.76 power = 0.99, and SDMT achievement rate: *p* < 0.001 ES(r) = 0.80 power = 0.99). 

### 3.4. Eye Movement Data

The median (interquartile range) of the driving image by VAS was 7.6 (6.8–8.4) for the older group and 7.6 (6.9–8.2) for the middle-aged group. Since there was no significant difference between the two groups, it was concluded that there was no difference in the effect of driving images on the eye movement data.

#### 3.4.1. Coordinates of the Gaze Point (X, Y)

Table 4 lists the results for the gazing coordinates. The mean, maximum, and minimum X-coordinates were not significantly different. The minimum Y-coordinates were not significantly different, but the mean and maximum were significantly different and significantly larger in the older group (*p* = 0.041 ES(r) = 0.39 power = 0.54, *p* =< 0.001 ES(r) = 0.74 power = 0.99, respectively). The larger the Y-coordinate, the lower the viewer’s gaze is on the monitor. In other words, the older group gazed closer at the car while watching the driving video.

#### 3.4.2. Total Number of Fixations

Table 5 shows the number of times the participants gazed at the screen during the driving videos. Significant differences were found in three of the ten scenes (Scenes 8, 9, and 10), with the older group gazing more frequently than the middle-aged group (*p* = 0.042 ES(r) = 0.38 power = 0.52, *p* = 0.043 ES(r) = 0.38 power = 0.52, *p* = 0.033 ES(r) = 0.40 power = 0.57, respectively). Scene 8 is a station roundabout scene, where one must watch out for people emerging from between stopped cars and getting out of cars. Scene 9 is a scene in which the driver makes a right turn at an intersection with no traffic signal and poor visibility while paying attention to many pedestrians walking on the roadway. Scene 10 is a scene in a parking lot of a large shopping center, where the driver must pay attention to the traffic of cars and pedestrians. In these three scenes, there was a lot of eye movement by the older drivers.

#### 3.4.3. Percentage of Participants Who Gazed at AOI

Out of a total of thirty-nine AOIs, five (2, 6, 16, 20, and 34) were excluded from the analysis because the percentage of drivers in the middle-aged group who gazed at them was less than 50.0%; therefore, it was highly likely that they were not recognized as hazard areas. The remaining 34 AOIs were included in the analysis. Only AOIs with significant differences were excerpted and the results are shown in Table 6.

The median (interquartile range) percentage of gazing at all AOIs was 77.9 (57.4–83.1)% and 91.2 (83.1–91.9)% for the older and middle-aged groups, respectively. This shows a significant difference between the two groups, with the older group having a smaller percentage of gazing (*p* = 0.011 ES(r) = 0.48 power = 0.76) than that of the middle-aged group. In other words, the older group is less likely to pay attention to the hazard area than the middle-aged group. Comparisons of the percentage of gazing at each AOI are shown in Table 5, with significant differences found at four AOIs (17, 21, 31, and 33), one of which (AOI 21) had a larger percentage of gazing in the older group (*p* = 0.044 ES(h) = 0.84 power = 0.61) than in the middle-aged group. AOI 21 depicts a pedestrian crossing near cars and was projected immediately after the video began. Three other locations (AOI 17, 31, and 33) had a smaller percentage of gazing by the older group (*p* = 0.018 ES(h) = 1.01 power = 0.75, *p* = 0.039 ES(h) = 0.84 power = 0.72, and *p* =< 0.001 ES(h) = 1.57 power = 0.98, respectively) than the middle-aged group. AOI 17 shows another car attempting to enter from behind a car turning left ahead, AOI 31 depicts a pedestrian right ahead in the roadway, and AOI 33 involves a curve mirror at an intersection with poor visibility and no signal.

#### 3.4.4. Total Duration of Fixation at AOI

The results of the comparison of the normalized cumulative gazing times for each AOI are listed in Table 7. Of the thirty-four AOIs, significant differences were found in nine AOIs (7, 10, 14, 17, 21, 31, 33, 35 and 38). At AOI 21, the older group had a longer gazing time than that of the middle-aged group (*p* = 0.029 ES(r) = 0.42 power = 0.62). AOI 21 depicts a pedestrian crossing near cars and was projected immediately after the video began. At eight other AOIs (7, 10, 14, 17, 31, 33, 35, 38), the older group had shorter gazing times than that of the middle-aged group (*p* = 0.042 ES(r) = 0.38 power = 0.52, *p* = 0.010 ES(r) = 0.48 power = 0.76, *p* = 0.017 ES(r) = 0.45 power = 0.69, *p* = 0.023 ES(r) = 0.48 power = 0.76, *p* = 0.013 ES(r) = 0.47 power = 0.74, *p* =< 0.001 ES(r) = 0.76 power = 0.99, *p* = 0.029 ES(r) = 0.42 power = 0.62, *p* = 0.013 ES(r) = 0.47 power = 0.74, respectively). AOI 7 includes another vehicle attempting to enter from the left side of the roadway; AOI 10 depicts a pedestrian crossing or sidewalk in the direction of a left turn; AOI 14 shows another car in the oncoming lane turning right at a scramble intersection; AOI 17 includes another car attempting to enter from behind a car turning left ahead; AOI 31 depicts a pedestrian ahead on the right side of the roadway; AOI 33 involves a curved mirror at an intersection with poor visibility and no signal; AOI 35 includes another car emerging from the right side of the roadway as it makes a right turn in the parking lot of a large shopping center; AOI 38 depicts an oncoming car passing another car in the opposite lane in the parking lot of a large shopping center.

#### 3.4.5. Time to First Fixation in AOI

Table 8 shows the results of the comparison of time to first gaze. For four (9, 17, 24, and 33) of the thirty-four AOIs, no statistical analysis was performed because of the small percentage of drivers in the older driver group who gazed (*n* < 7). Of the remaining thirty AOIs, significant differences were found at two AOIs (4 and 14), with the older group taking longer to look for the first time than the middle-aged group (*p* = 0.016 ES(r) = 0.45 power = 0.69, *p* = 0.042 ES(r) = 0.38 power = 0.52, respectively). AOI 4 depicts another car trying to enter from the parking lot ahead on the left, and AOI 14 shows another car in the oncoming lane turning right at a scramble intersection.

#### 3.4.6. Number of Visits to AOI 

Table 9 presents the results of the normalized number of visits to an AOI. Out of the thirty-four AOIs, significant differences were found in nine AOIs (5, 10, 17, 18, 30, 31, 33, 35, and 38), with the older group having fewer visits than the middle-aged group (*p* = 0.026 ES(r) = 0.42 power = 0.61, *p* = 0.037 ES(r) = 0.40 power = 0.57, *p* = 0.010 ES(r) = 0.55 power = 0.89, *p* = 0.042 ES(r) = 0.38 power = 0.52, *p* = 0.047 ES(r) = 0.37 power = 0.49, *p* = 0.026 ES(r) = 0.43 power = 0.64, *p* =< 0.001 ES(r) = 0.76 power = 0.99, *p* = 0.017 ES(r) = 0.45 power = 0.69, *p* = 0.037 ES(r) = 0.39 power = 0.54, respectively). AOI 5 depicts a pedestrian crossing far ahead; AOI 10 depicts a pedestrian crossing or sidewalk in the direction of a left turn; AOI 17 involves another car attempting to enter from behind a car turning left ahead; AOI 18 involves being in the shadow (blind spot) of a parked construction vehicle; AOI 30 depicts a pedestrian in front of the left side of the roadway; AOI 31 depicts a pedestrian in front of the right side of the roadway; AOI 33 includes a curved mirror at an intersection with poor visibility and no signal; AOI 35 involves another car emerging from the right side of the roadway as it makes a right turn in the parking lot of a large shopping center; and AOI 38 shows an oncoming car passing another car in the opposite lane in the parking lot of a large shopping center.

#### 3.4.7. Summary of Parameters for AOI

The analyses revealed significant differences in 13 of the 34 total AOIs (AOIs 4, 5, 7, 10, 14, 17, 18, 21, 30, 21, 33, 35, and 38). A summary of the hazard type, direction of travel, and gaze parameters for the older group for each AOI is shown in Table 10. Hazard types were divided into two types: road environment and road users [30,71]. The road environment includes signs, buildings, parked cars, pedestrian crossings, railroad crossings, and road users include cars, pedestrians, bicycles [71]. The roadway environment includes distant pedestrian crossings (AOI 5), left turn entrapment (AOI 10), blind spots of stopped vehicles (AOI 18), curve mirrors at intersections with poor visibility and no signals (AOI 33), and pedestrian crossings nearby (AOI 21). Road users included cars entering the roadway (AOI 4, 7, 17), oncoming cars turning right (AOI 14), pedestrians walking on the roadway (AOI 30, 31), other cars at intersections (AOI 35), and oncoming cars when overtaking (AOI 38). There were eight straight-ahead scenes (AOIs 4, 5, 7, 17, 18, 30, 31, and 38) and five right/left turn scenes (AOIs 10, 14, 21, 33, and 35).

Of these AOIs, AOI 21 was the only one that differed, showing more driver gazing and longer gazing times for older drivers than for middle-aged drivers. The other 12 AOIs (4, 5, 7, 10, 14, 17, 18, 30, 21, 33, 35, 38) showed fewer/shorter values in one or more of the following items: percentage of gazing, gazing time, time to gaze, and number of visits. AOI 30, 31, 33, 35, and 38 are danger areas included in Scenes 9 and 10; despite a large amount of eye movement when viewing the driving video, gazing time and number of visits were not significant.

## 4. Discussion

We analyzed the gaze of older drivers while watching driving videos to clarify their gazing characteristics whilst driving a car. Older and middle-aged drivers who live and drive daily in a certain automobile-dependent area were included in the study. Middle-aged people, who are considered to be skilled drivers and the age group with the best hazard prediction, were set as the control group.

### 4.1. Gazing Characteristics in Driving

The results of coordinates of the gaze point showed that there were no significant differences in the mean, maximum, and minimum horizontal gaze coordinates. Previous studies have shown that as driving experience increases, drivers perceive hazards based on similar episodes in the past. Therefore, they gaze at locations where potential hazards are more likely to appear [74] and search along the horizontal axis parallel to the road where potential hazards are more likely to appear [75]. The older drivers in this study had nearly half a century of driving experience, and their extensive driving experience likely demonstrated a broad horizontal search while considering potential hazards, with a search range that appears to be comparable to that of the middle-aged group. However, regarding the vertical gazing coordinates, significant differences were found in the mean and maximum, but not in the minimum. It is thought that although the respondents gazed at locations away from the car, older adults tended to gaze at locations closer to the car as a whole. This may indicate a spread of search in the vertical direction. Downward eye movements are less affected by aging [59], which may reflect the characteristic that older drivers are more likely to gaze downward. Underwood [75] and Maurant and Rockwell [76] have stated that inexperienced drivers with poor hazard prediction search along vertical directions or concentrate on roads close to their vehicles. This trend is similar to that of walking and bicycling [77,78] and can be considered to be common among the older adults when they move around. The fact that the older group in this study searched in the proximate area of their car indicates a spread of vertical search, which may indicate a decline in judgment and the ability to predict hazards due to aging.

The results of the total number of fixations showed that the older group had more eye movements in three scenes (8, 9 and 10) than the middle-aged group. These scenes are considered to have required extensive attention, especially because they are situations that require driving with consideration for road users. Although the vertical search spread of gazing coordinates may be responsible for the greater amount of eye movement while watching driving videos, the lack of significant differences between the two groups in many scenes suggests that eye movement during driving is comparable between the different age groups.

### 4.2. Gaze Characteristics for Hazard Areas

Regarding the percentage of participants who gazed at an AOI, the older group was less likely than the middle-aged group to have gazed at the entire hazard area, suggesting that the older group may have a reduced ability to perceive hazards and be more likely to miss hazards. Comparison results for each AOI showed that the percentage of gazing was low at only three AOIs (17, 31, and 33). Overall, it seems unlikely that the older group would have such a significantly reduced hazard detection ability that it would interfere with safe driving.

Regarding total fixation time at an AOI, there were significant differences at nine of the AOIs. Although prolonged gazing time is an indicator of decreased cognitive processing speed [38], only one AOI (AOI 21) showed prolonged gazing time, suggesting that cognitive processing speed in the older group was not decreased. However, regarding the short gazing time in the other eight AOIs (7, 10, 14, 17, 31, 33, 35, and 38), it is questionable whether the older drivers were able to make accurate judgments and recognize situations with shorter gazing times than the middle-aged group, considering the significantly lower performance of the younger group in the cognitive and attentional function test results. Carid et al. [79] have stated that “gazing at a location does not necessarily imply that the visual information is cognitively processed,” especially in older drivers. In particular, they identify that it is difficult to accurately scan all objects in a limited amount of time, particularly when potential hazards occur simultaneously or at complex intersections [79]. Considering that AOI 14, 17, 31, 33, 35, and 38 are complex road environments with multiple hazards such as other cars, pedestrians, pedestrian crossings, intersections, traffic signals, and road signs, a short normalized cumulative gazing time may indicate poor cognitive processing rather than good hazard prediction.

Regarding time to first fixation on an AOI, although the older driver group had a slower time to fixation in two of thirty AOIs (AOI 4 and 14), many AOIs were not significantly different from the middle-aged group, suggesting that the reaction time to the hazard of the older group is not slow. This result differs from previous reports [26,80] which show that the older one gets, the slower one’s reaction time response to hazard prediction. The discrepancy may be because the method used by Horswill et al. to evaluate reaction speed was the act of pushing a button. However, statistical treatment was not performed for four AOIs (9, 17, 24, and 33) due to the small number of older drivers who gazed at the AOI. In addition, the results of the percentage of participants who gazed at AOIs showed that the older group was likely to miss the hazard.

Regarding the number of visits to an AOI, the older group had fewer visits in nine AOIs (5, 10, 17, 18, 30, 31, 33, 35 and 38) than the middle-aged group. Given that drivers with better hazard prediction frequently watch for hazardous locations and therefore make more visits [39], it was suggested that the hazard prediction of the older group may be reduced.

### 4.3. Traffic Situation of Each AOI

Significant differences were found in 13 of the 34 AOIs (AOIs 4, 5, 7, 10, 14, 17, 18, 21, 30, 21, 33, 35, and 38). Of these, only the closest crosswalk (AOI 21) had a higher gazing rate and longer gazing time, which is a sign of how the gazing coordinates of the older group extended closer to the car. In both straight-going scenes and right/left turning scenes, negative results were observed regarding fixation percentage, fixation duration, reaction time, or number of visits, concerning road environments such as distant pedestrian crossings (AOI5), potential collision areas during left turns (AOI10), blind spots of parked vehicles (AOI18), and curved mirrors at intersections (AOI33), as well as road users such as intruding cars (AOI4, 7, and 17), oncoming cars during right turns (AOI14), pedestrians (AOI 30 and 31), overtaking cars (AOI 35), and other cars at intersections (AOI 38). Therefore, the older group may have insufficient consideration for the danger of other vehicles and pedestrians entering the roadway. In particular, AOIs 5, 10, and 18 are not realized hazard situations but rather potential hazard locations where pedestrians and vehicles may jump out of the way. This is not contradictory to the report by Underwood et al. [81], which states that middle-aged experienced drivers focus more on areas where potential dangers are more likely to occur compared to young or older drivers. Furthermore, these results are consistent with reports [23,25,29] that older drivers are less likely to check the traffic situation when turning left or right. AOIs 30, 31, 33, 35, and 38 are scenes where visibility is poor and there are many road users and, therefore, careful driving is required. However, the number of visits to AOIs is small and the fixation duration on AOIs is short. The older group may not have been gazing more frequently at non-hazardous rather than hazardous areas. This result is similar to those reported by Scott-Parker et al. [18] and Caird et al. [78], in which older drivers were more likely to gaze at less hazardous areas of the road environment. Scott-Parker et al. [18] identify that older drivers are distracted while driving; therefore, the frequent gazing may be due to distraction. Older drivers change their driving performance in more complex situations, such as changing lanes, turning left at unsignaled intersections, and merging onto highways [82]. They may be likely to exhibit inefficient scanning patterns, overlook hazards, or be slow to react in traffic situations with complex road users and road environments.

### 4.4. Limitations

Owing to this study’s small sample size, few of the gaze parameters that were statistically significant had high power, even though the effect sizes were all above moderate. This limits the interpretation of the significance. Future studies should use larger sample sizes to improve the power and reliably validate the results.

Second, age-related changes in eye movements observed in the laboratory may not fully translate to the real-world scenario [83], thus limiting their ecological validity. Chapman and Underwood [35] and Underwood et al. [84] have established that video-showing tasks are sensitive to driving-related skills. However, while watching the video, the participants did not need to control the vehicle; therefore, the task had a low difficulty level. It should be noted that the performance might differ from the actual control of the car. The extent to which the video-based tasks used in this study relate to actual driving behavior is unclear. In addition, for visibility and analysis accuracy, this study used videos in a brightly lit environment during the daytime. However, future analyses should also include scenes at night and during dark hours.

In actual driving situations, older drivers attempt to compensate for their decline in cognitive and motor functions at a behavioral level, such as driving at slower speeds, adhering to long following distances, and leaving large gaps at intersections [45,65,66,67,82]. Since the driving video used in this study was a recording of someone else’s driving, the uncomfortable driving speed and behavior may have affected the results of eye movement data for older adults. Furthermore, the characteristics of visual attention in older adult drivers suggested in this study may reflect risk processing methods to compensate for cognitive decline, reduced visual attention, and reduced efficiency of visual search. However, the lack of direct data on specific risk perception and processing methods makes this a matter of speculation. In the future, we hope that quantitative and qualitative analyses of drivers’ risk processing methods will lead to more reliable conclusions. Such a method would help clarify the differences according to age. In addition, pharmacological treatment of age-related conditions such as stroke and diabetes can cause side effects, which can also affect driving safety [85]. The use of drugs for common diseases may affect hazard prediction. Future studies should consider study designs that allow for more diverse information to be collected, as well as more detailed data on participants’ health status and drug use.

## 5. Conclusions

This is a pilot study in which an eye gaze analysis was conducted on older and middle-aged drivers while they viewed automobile driving videos. While older adult drivers show the same horizontal search range as middle-aged drivers when driving a car, their search expands in the vertical direction, particularly close to the car, and their gaze shifts in more complex traffic situations. This is likely to increase. There is a possibility that hazard detection ability is reduced for older adults. Rather than a delay in reaction time to hazards, it seems likely that this is due to overlooked hazards. In addition, although the cognitive processing speed for hazards did not show a significant decrease, it is likely that the time and frequency required for the cognitive processing of hazards were insufficient. Therefore, it is possible that accurate cognitive processing may not be occurring. In particular, in situations where careful driving is required, older adults exhibit a lot of eye movement; however, they gaze at unimportant areas, which shows an inefficient scanning pattern.

Although the results of this study suggest that a decrease in hazard prediction may be apparent in some situations, these findings are only a partial result of gaze parameters and do not indicate a clear decline in driving ability in older drivers. Some older drivers avoid driving under adverse conditions (e.g., bad weather, nighttime, and congested or complicated traffic conditions) as a safety strategy [86,87]. It is also important to suggest self-regulation of driving behavior to avoid the risk of causing an accident by adjusting vehicle speed and driving distance, driving during the day, and avoiding complex traffic and road conditions to compensate for age-related decline [87]. Furthermore, Horswill et al. [14,26] have identified the need for the assessment and training of older drivers regarding their ability to predict hazards. Interventions aimed at improving the hazard prediction ability and the development of cars to compensate for the reduced hazard prediction ability should be pursued.

## Figures and Tables

**Table 1:** Background of the participants.

	Older	Middle-Aged
age (year)	73.9 (3.2)	37.3 (2.9)
driving history (year)	49.1 (9.7)	18.0 (2.4)
nude/contact lens/glasses (n)	12/1/3	4/5/3
eye surgery (yes/no)	2/14	2/10

Mean (standard deviation).

**Table 2:** Results for basic attributes.

	Older	Middle-Aged	*p*-Value
male/female (n)	3/13	5/7	n.s.
driving frequency (count/week)	7 (3–7)	7 (7–7)	n.s.
driving time (min/week)	50 (30.0–60.0)	40 (30.0–67.5)	n.s.
experience of traffic violations (%)	18.7	0.0	n.s.
experience of traffic crashes (%)	12.5	8.3	n.s.

Median (interquartile range); Fisher’s exact test and Mann–Whitney U test; n.s., no significant difference.

**Table 3:** Results for cognitive function and attention function.

	Older	Middle-Aged	*p*-Value	ES(r)	Power Value
MMSE (scores)	29.0 (27.8–30.0)	30.0 (30.0–30.0)	0.002	0.58	0.93
TMT-A (s)	46.6 (41.7–65.7)	31.0 (23.1–34.8)	<0.001	0.72	0.99
TMT-B (s)	89.3 (75.8–113.6)	39.3 (34.1–43.7)	<0.001	0.76	0.99
SDMT (%)	37.3 (34.3–43.8)	59.0 (55.0–64.7)	<0.001	0.80	0.99

Median (interquartile range); Mann–Whitney U test; MMSE, Mini-Mental State Examination; TMT-A, Trail Making Test Part A; TMT-B, Trail Making Test Part B; SDMT, Symbol Digit Modalities Test. ES(r), effect size(r); approximate amount of effect size, small; >0.10, medium; >0.30, large; >0.50.

**Table 4:** Coordinates of the gaze point.

		Older	Middle-Aged	*p*-Value	ES(r)	Power
mean (%)	X	52.2 (52.0–53.1)	52.1 (51.5–52.4)	n.s.	0.20	0.17
	Y	59.6 (57.9–61.7)	57.9 (56.5–58.9)	0.041	0.39	0.54
max (%)	X	73.3 (71.7–78.1)	76.0 (74.1–76.9)	n.s.	0.14	0.10
	Y	85.3 (83.6–90.9)	75.9 (71.8–78.1)	<0.001	0.74	0.99
min (%)	X	28.9 (24.3–32.0)	27.2 (23.8–28.7)	n.s.	0.24	0.22
	Y	44.2 (39.1–47.8)	43.5 (41.5–45.1)	n.s.	0.11	0.08

Median (interquartile range); Mann–Whitney U test; n.s., no significant difference. X: The horizontal midline is 50%, and 50% or less indicates gazing to the left, while 50% or more indicates gazing to the right. Y: Vertical midline is 50%, and 50% or less indicates gazing upwards, while 50% or more indicates gazing downwards. ES(r), effect size(r); approximate amount of effect size, small; >0.10, medium; >0.30, large; >0.50.

**Table 5:** Total number of fixations.

	Older	Middle-Aged	*p*-Value	ES(r)	Power
Scene 8 (count)	63.5 (57.8–71.0)	57.0 (48.5–61.0)	0.042	0.38	0.52
Scene 9 (count)	60.5 (52.5–63.8)	51.5 (46.5–55.0)	0.043	0.38	0.52
Scene 10 (count)	67.0 (60.3–73.0)	57.5 (53.0–62.3)	0.033	0.40	0.57

Median (interquartile range); Mann–Whitney U test. ES(r), effect size(r); approximate amount of effect size, small; >0.10, medium; >0.30, large; >0.50.

**Table 6:** Percentage of participants who gazed at the area of interest.

	Traffic Situation	Older	Middle-Aged	*p*-Value	ES(h)	Power
AOI 17(%) (Scene 5)	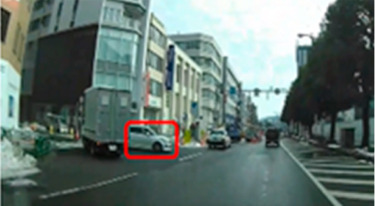	18.7	66.6	0.018	1.01	0.75
AOI 21(%) (Scene 7)	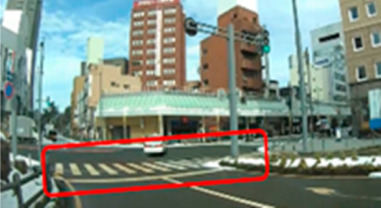	87.5	50.0	0.044	0.84	0.61
AOI 31(%) (Scene 9)	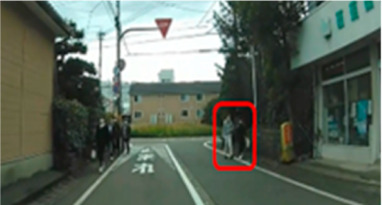	50.0	91.6	0.039	0.98	0.72
AOI 33 (%) (Scene 9)	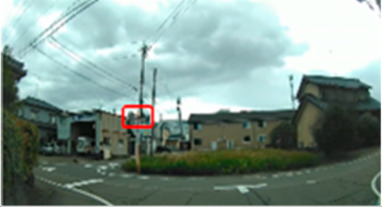	12.5	83.3	<0.001	1.57	0.98

Fisher’s exact test; red frame = area of interest (AOI). ES(h), effect size (Cohen-h); approximate amount of effect size, small; >0.20, medium; >0.50, large; >0.80.

**Table 7:** Total duration of fixation in the area of interest.

	Traffic Situation	Older	Middle-Aged	*p*-Value	ES(r)	Power
AOI 7(%) (Scene 2)	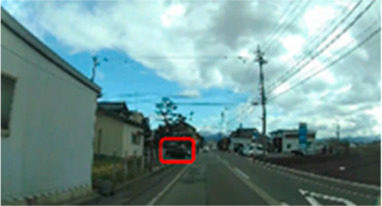	7.8 (0.0–19.7)	22.8 (13.0–31.0)	0.042	0.38	0.52
AOI 10(%) (Scene 3)	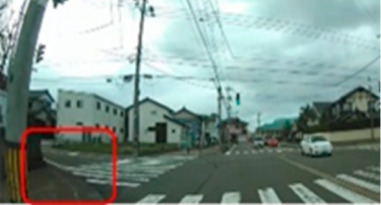	11.3 (0.0–28.5)	45.0 (26.8–51.5)	0.010	0.48	0.76
AOI 14(%) (Scene 4)	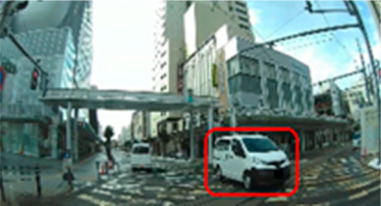	43.8 (25.2–52.8)	60.7 (43.9–74.8)	0.017	0.45	0.69
AOI 17(%) (Scene 5)	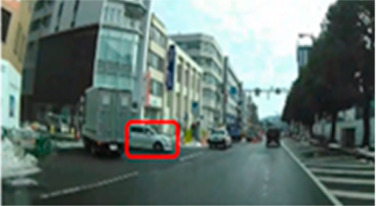	0.0 (0.0–0.0)	3.6 (0.0–11.4)	0.023	0.48	0.76
AOI 21(%) (Scene 7)	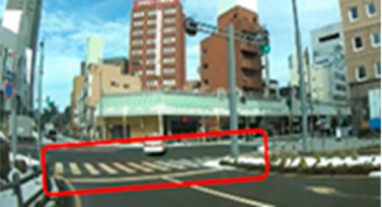	19.4 (7.6–47.1)	2.0 (0.0–23.9)	0.029	0.42	0.62
AOI 31(%) (Scene 9)	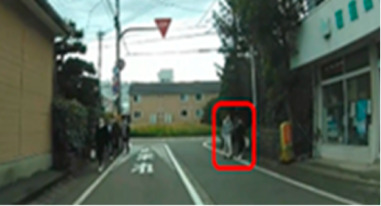	0.9 (0.0–4.9)	7.4 (3.7–10.0)	0.013	0.47	0.74
AOI 33(%) (Scene 9)	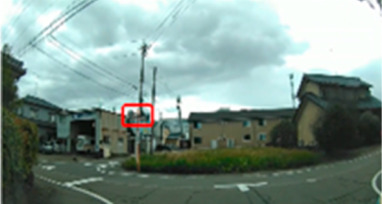	0.0 (0.0–0.0)	30.0 (14.2–43.4)	<0.001	0.76	0.99
AOI 35(%) (Scene 10)	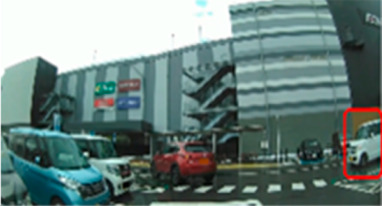	18.8 (0.0–13.7)	23.7 (11.8–34.8)	0.029	0.42	0.62
AOI 38 (%) (Scene 10)	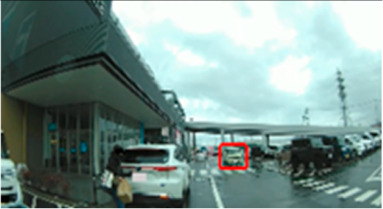	6.1 (4.2–15.1)	24.8 (10.5–37.0)	0.013	0.47	0.74

Median (interquartile range); Mann–Whitney U test; red frame = area of interest (AOI). ES(r), effect size(r); approximate amount of effect size, small; >0.10, medium; >0.30, large; >0.50.

**Table 8:** Time to first fixation on the area of interest.

	Traffic Situation	Older	Middle-Aged	*p*-Value	ES(r)	Power
AOI 4(sec) (Scene1)	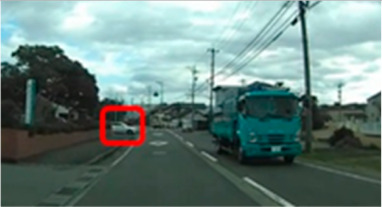	18.1 (17.9–21.4)	15.9 (15.9–17.9)	0.016	0.45	0.69
AOI 14(sec) (Scene4)	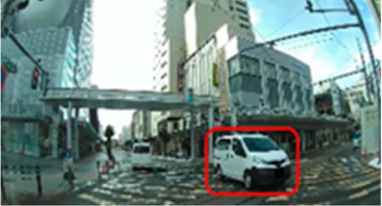	5.1 (3.4–5.4)	3.5 (2.8–4.1)	0.042	0.38	0.52

Median (interquartile range); Mann–Whitney U test; red frame = area of Interest (AOI). ES(r), effect size(r); approximate amount of effect size, small; >0.10, medium; >0.30, large; >0.50.

**Table 9:** Number of visits in the area of interest.

	Traffic Situation	Older	Middle-Aged	*p*-Value	ES(r)	Power
AOI 5(%) (Scene 1)	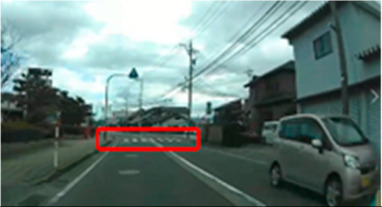	6.1 (3.6–7.7)	8.8 (6.5–11.1)	0.026	0.42	0.61
AOI 10(%) (Scene 3)	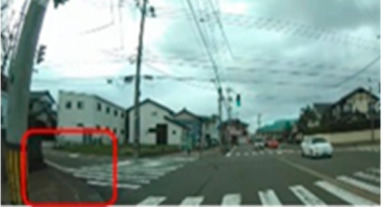	2.9 (0.0–4.0)	4.5 (3.6–5.8)	0.037	0.40	0.57
AOI 17(%) (Scene 5)	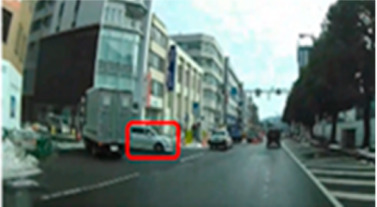	0.0 (0.0–0.0)	2.6 (0.0–3.5)	0.010	0.55	0.89
AOI 18(%) (Scene 5)	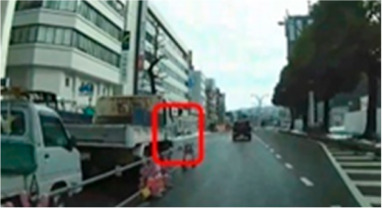	5.0 (3.3–6.8)	7.8 (4.8–8.8)	0.042	0.38	0.52
AOI 30(%) (Scene 9)	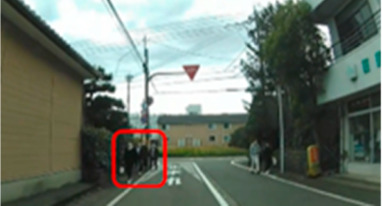	6.1 (3.8–8.2)	8.0 (6.6–10.0)	0.047	0.37	0.49
AOI 31(%) (Scene 9)	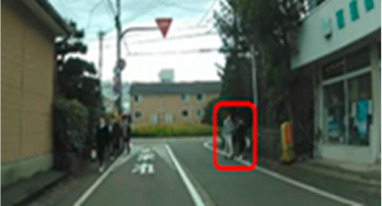	0.7 (0.0–2.4)	2.8 (2.1–4.2)	0.026	0.43	0.64
AOI 33(%) (Scene 9)	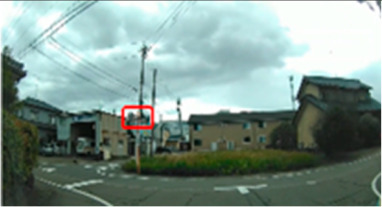	0.0 (0.0–0.0)	4.1 (2.1–6.6)	<0.001	0.76	0.99
AOI 35(%) (Scene 10)	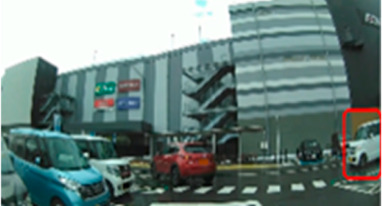	0.7 (0.0–2.4)	3.3 (1.6–3.8)	0.017	0.45	0.69
AOI 38(%) (Scene 10)	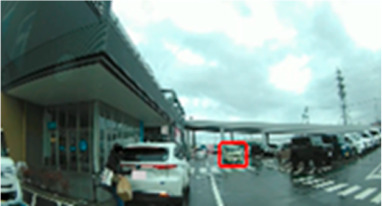	3.8 (1.5–7.4)	7.7 (5.2–8.4)	0.037	0.39	0.54

Median (interquartile range); Mann–Whitney U test; red frame = area of interest (AOI). ES(r), effect size(r); approximate amount of effect size, small; >0.10, medium; >0.30, large; >0.50.

**Table 10:** Summary of parameters for the area of interest.

AOI	Hazard	Direction	Fixations	Percentage	Duration	Time	Visits
4 (Scene1)	users	straight				slow	
5 (Scene1)	environment	straight					few
7 (Scene2)	users	straight			short		
10 (Scene3)	environment	left turn			short		few
14 (Scene4)	users	right turn			short	slow	
17 (Scene5)	users	straight		small	short		few
18 (Scene5)	environment	straight					few
21 (Scene7)	environment	left turn		large	long		
30 (Scene9)	users	straight	many				few
31 (Scene9)	users	straight	many	small	short		few
33 (Scene9)	environment	right turn	many	small	short		few
35 (Scene10)	users	right turn	many		short		few
38 (Scene10)	users	straight	many		short		few

AOI, area of interest; fixations = total number of fixations; percentage = percentage of participants who gazed at the AOI; duration = total duration of fixation at the AOI; time = time to first fixation in the AOI; visits = number of visits in the AOI. Underline: ES (r) > 0.50, power > 0.80.

## Data Availability

The data presented in this study are available upon request from the corresponding author. The data are not publicly available because of privacy concerns.
